# The Association Between Breast Cancer and Blood-Based Methylation of *CD160*, *ISYNA1* and *RAD51B* in the Chinese Population

**DOI:** 10.3389/fgene.2022.927519

**Published:** 2022-06-09

**Authors:** Chunlan Liu, Xiajie Zhou, Jialie Jin, Qiang Zhu, Lixi Li, Qiming Yin, Tian Xu, Wanjian Gu, Fei Ma, Rongxi Yang

**Affiliations:** ^1^ Department of Epidemiology, School of Public Health, Nanjing Medical University, Nanjing, China; ^2^ Department of Medical Oncology, National Cancer Center/National Clinical Research Center for Cancer/Cancer Hospital, Chinese Academy of Medical Sciences & Peking Union Medical College, Beijing, China; ^3^ Department of Clinical Laboratory, Jiangsu Province Hospital of Chinese Medicine, Nanjing, China

**Keywords:** breast cancer, DNA methylation, cluster of differentiation 160 gene, inositol-3-phosphate synthase 1 gene, RAD51 paralog B gene

## Abstract

Recent studies have identified DNA methylation signatures in the white blood cells as potential biomarkers for breast cancer (BC) in the European population. Here, we investigated the association between BC and blood-based methylation of cluster of differentiation 160 (*CD160*), inositol-3-phosphate synthase 1 (*ISYNA1*) and RAD51 paralog B (*RAD51B*) genes in the Chinese population. Peripheral blood samples were collected from two independent case-control studies with a total of 272 sporadic early-stage BC cases (76.5% at stage I&II) and 272 cancer-free female controls. Mass spectrometry was applied to quantitatively measure the levels of DNA methylation. The logistic regression and non-parametric tests were used for the statistical analyses. In contrast to the protective effects reported in European women, we reported the blood-based hypomethylation in *CD160*, *ISYNA1* and *RAD51B* as risk factors for BC in the Chinese population (CD160_CpG_3, CD160_CpG_4/cg20975414, ISYNA1_CpG_2, RAD51B_CpG_3 and RAD51B_CpG_4; odds ratios (ORs) per -10% methylation ranging from 1.08 to 1.67, *p* < 0.05 for all). Moreover, hypomethylation of *CD160*, *ISYNA1* and *RAD51B* was significantly correlated with age, BC subtypes including estrogen receptor (ER)-negative BC tumors, triple negative tumors, BC cases with larger size, advanced stages and more lymph node involvement. Our results supported the report in European women that BC is associated with altered methylation of *CD160*, *ISYNA1* and *RAD51B* in the peripheral blood, although the effects are opposite in the Chinese population. The difference between the two populations may be due to variant genetic background or life styles, implicating that the validations of epigenetic biomarkers in variant ethnic groups are warranted.

## Introduction

Breast cancer (BC) is the most commonly diagnosed cancer among women worldwide, with an estimated 2.3 million new cases in 2020 (Sung et al., 2021). Despite therapeutic advances in chemotherapy, radiotherapy, hormone and targeted therapies, BC remains the leading cause of cancer mortality among females globally (Sung et al., 2021). Mammography is currently the most widely-used screening tool for the detection of BC, which is estimated to decrease BC mortality by 20–40% (Berry et al., 2005; Seely and Alhassan, 2018). However, the benefit of mammography for women aged 40 to 49 with dense breast tissue is uncertain (Moss et al., 2015). In addition, the radiological exposure, false-positive results and overdiagnosis are limitations of concern (Independent United Kingdom Panel on Breast Cancer Screening, 2012; Pace and Keating, 2014). Thus, the identification of new reliable markers for the early detection and/or risk stratification of BC is urgently needed.

Epigenetic modifications are heritable and can alter gene expression without changes in the DNA sequence (Jones and Baylin, 2002; Feinberg, 2004). Epigenetic abnormalities, particularly aberrant DNA methylation events, are critical factors for the initiation and progression of human cancers (Jones and Baylin, 2002; Grady et al., 2021). Hypermethylation in the promoter regions of tumor suppressor genes and global hypomethylation have been recognized as the early events in almost every cancer type such as breast (Umbricht et al., 2001), lung (Belinsky et al., 1998), and colon cancer (Rademakers et al., 2021). Previous studies have found DNA methylation alterations in circulating free DNA (cfDNA) of BC patients (Fackler et al., 2014; Mahon et al., 2014; Sun et al., 2015; Matsui et al., 2016; Uehiro et al., 2016). Recently, several investigations have identified DNA methylation signatures in the white blood cells as potential biomarkers for the detection of BC, but were mostly limited by low statistical power (Iwamoto et al., 2011; Brennan et al., 2012; Cappetta et al., 2021). Yang et al. (Yang et al., 2020) have developed a new methodology using genome-wide association study (GWAS) data to evaluate the DNA methylation levels at adjacent CpG sites, and identified 450 BC-associated CpGs. By integrative analysis of genetic variations, DNA methylation and gene expression data, they found that 38 CpGs in 21 genes could affect BC via regulating gene expression. Since DNA methylation patterns are influenced by genetic backgrounds or life styles (Zhang et al., 2011; Wu et al., 2020; Rasmussen et al., 2021), it would be meaningful to validate the associations between BC and methylation of these 21 genes in other ethnic groups especially via a quantitative assay. Hereby, we performed MassARRAY to quantitatively evaluate methylation-altered genes in the peripheral blood DNA that are associated with BC risk in two independent case-control studies with a total of 272 sporadic BC cases and 272 cancer-free female controls in the Chinese population.

## Materials and Methods

### Study Population

This study was approved by the Ethics Committee of Nanjing Medical University, the Cancer Hospital of Chinese Academy of Medical Science and Jiangsu Province Hospital of Chinese Medicine in China. All the recruited participants provided written informed consent. The diagnosis of BC was confirmed by pathology, and all peripheral blood samples were collected before surgery and any BC related treatment. All the female controls have normal blood cell counts and have claimed no history of tumor or autoimmune disease. No further inclusion criteria were applied during recruitment of controls.

Validation I: A total of 48 sporadic BC cases with a median age of 44 years (34–50 years old) were collected at the Cancer Hospital of Chinese Academy of Medical Science from 2015 to 2018. Forty-eight age-matched female controls (median age: 44 years, range from 28 to 65 years) were consecutively collected from the physical examination center at Jiangsu Province Hospital of Chinese Medicine in 2018.

Validation II: A total of 224 sporadic BC cases with a median age of 46 years (35–73 years old) were collected at the Cancer Hospital of Chinese Academy of Medical Science from 2015 to 2018. In addition, 224 age-matched cancer-free female controls (median age: 46 years, range from 25 to 78 years) were randomly recruited from the physical examination center at Jiangsu Province Hospital of Chinese Medicine in 2018.

### Sample Processing and Bisulfite Conversion

Peripheral blood samples from BC cases and controls were collected by ethylene diamine tetraacetic acid (EDTA) tubes. The blood samples were kept at 4°C for less than 24 h and further stored at −80°C till usage. Genomic DNA was isolated from peripheral whole blood using the DNA Extraction Kit (TANTICA, Nanjing, China), and then bisulfite converted by EZ-96 DNA Methylation Gold Kit (Zymo Research, Orange County, United States) according to the manufacturer’s protocol. After bisulfite treatment, all non-methylated cytosine (C) bases in CpG sites were converted to uracil (U), whereas all methylated C bases remained C. The samples from BC cases and controls were processed in parallel.

### Selection of Methylated Gene Markers

**FIGURE 1:**
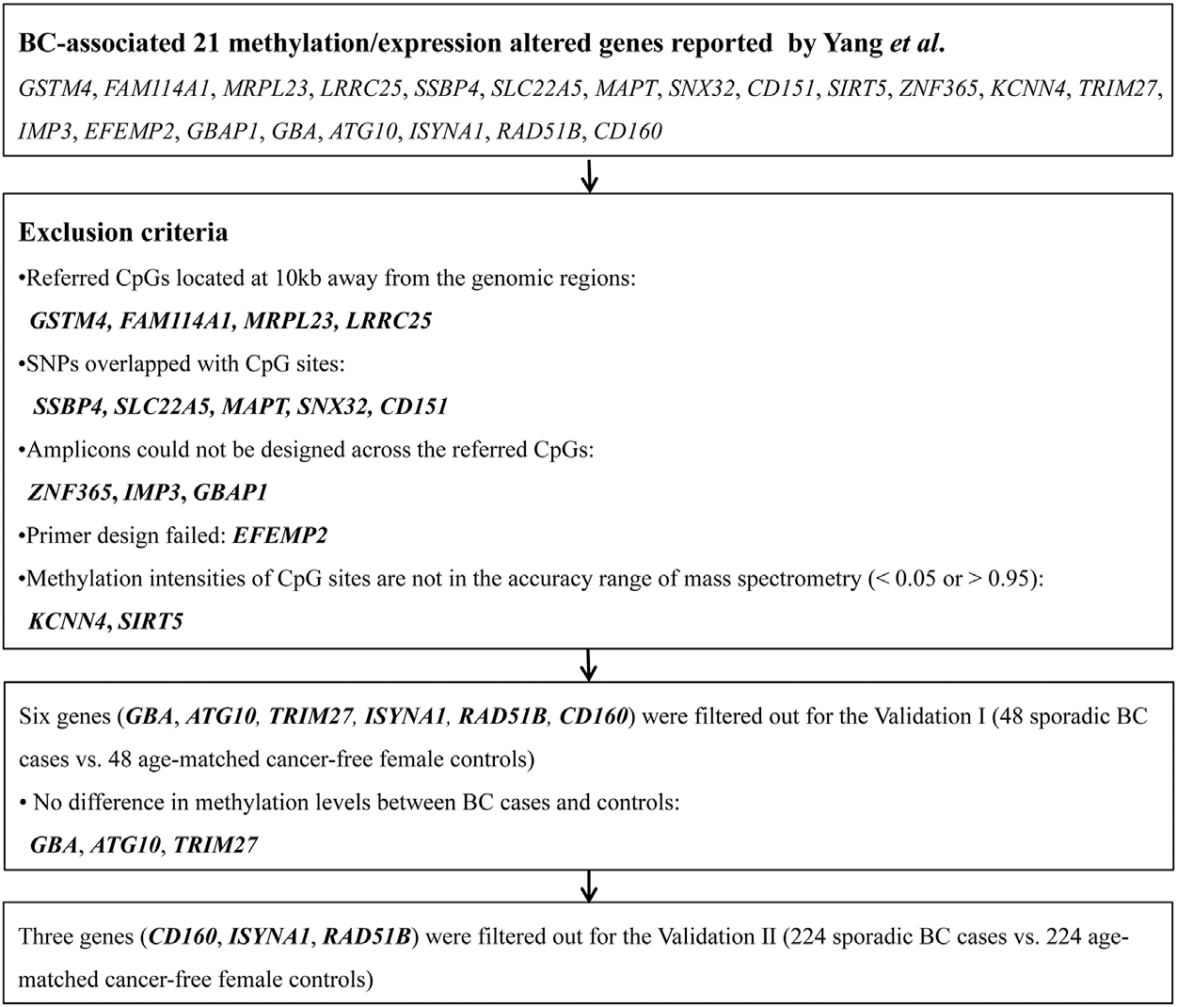
Work-flow for the selection of methylated genes. The flowchart shows the steps to filter out the altered DNA methylation markers for the validation via mass spectrometry in the Chinese population.

To validate the 21 methylation-altered genes that are associated with BC risk reported by Yang et al. (Yang et al., 2020), we applied the following exclusion criteria to select the CpG sites: 1) referred CpGs located at 10 kb away from the genomic regions; 2) single nucleotide polymorphisms (SNPs) overlapped with CpG sites; 3) amplicons could not be designed across the referred CpGs; 4) primer design failed; and 5) methylation intensities of CpG sites are not in the accuracy range of mass spectrometry (methylation intensity <0.05 or >0.95) (Figure 1). Six genes (*GBA*, *ATG10*, *TRIM27*, *CD160*, *ISYNA1*, *RAD51B*) were filtered out for the validation I with 48 sporadic BC cases and 48 matched cancer-free female controls. Three genes (*CD160*, *ISYNA1*, *RAD51B*) that showed significant difference in methylation levels between BC cases and controls in validation I were further filtered out for the validation II with 224 sporadic BC cases and 224 matched female controls (Figure 1).

### Matrix-Assisted Laser Desorption/Ionization Time-Of-Flight Mass Spectrometry

The methylation levels of CpG sites were quantitatively determined by MALDI-TOF mass spectrometry (Agena Bioscience, San Diego, California, United States). The cg20975414 (chr1: 145,715,572) and cg12832565 (chr1: 145,715,673) loci in *CD160* gene reported by Yang et al. are located at the promoter region of *CD160*. We therefore designed an amplicon (498 bp, chr1: 145,715,317–145,715,815) harboring both cg20975414 and cg12832565 loci and flanking four CpG sites. This amplicon covers the promoter region and exon 1 of *CD160* gene, as well as part of intron 1. The cg22161383 (chr19: 18,545,441) in *ISYNA1* gene reported by Yang et al. is located at the exon 8 of *ISYNA1*. An amplicon (454 bp, chr19: 18,545,150–18,545,604, at the exon 8 of *ISYNA1*) harboring cg22161383 and flanking six CpG sites was designed. The cg13803234 (chr14: 68,830,813) and cg10975863 (chr14: 68,830,704) loci in *RAD51B* gene reported by Yang et al. are located at intron 8 of *RAD51B*. We therefore designed an amplicon (478 bp, chr14: 68,830,515–68,830,993, at intron 8 of *RAD51B*) covering both cg13803234 and cg10975863. The sequences of amplicons are presented in Supplementary Figure S1. Briefly, the bisulfite-converted genomic DNA was amplified by bisulfite-specific primers. There were no SNPs located at the primer regions or overlapped with the CpG sites. Following shrimp alkaline phosphatase cleanup, T cleavage, and Clean Resin steps, the final products were transferred to a SpectroCHIP G384 by a Nanodispenser RS1000 apparatus (Agena, United States) and then the chips were detected by MassARRAY spectrometry. The quantitative methylation levels of each CpG site or aggregate of multiple CpG sites were collected by SpectroACQUIRE v3.3.1.3 software and visualized by EpiTyper v1.3 software. The EpiTyper v1.3 software automatically calculate methylation levels of each CpG locus in the investigated amplicon by comparing the signal intensities of methylated and non-methylated segments.

### Statistical Analyses

All the statistical data were analyzed using SPSS 25.0 software (SPSS Inc., Chicago, United States). The individual CpG site differences between two or three groups were assessed by non-parametric tests. The methylation differences between the cases and controls were analyzed by binary logistic regression. The dependent variable was the status of existence of disease (case = 1, control = 0), the independent variable was the DNA methylation levels of each CpG site. The covariables were adjusted, including age and batches of different measurements. A two-tailed *p* value <0.05 was considered statistically significant.

## Results

### Validation of Breast Cancer-Associated DNA Methylation Markers in the Peripheral Blood

**FIGURE 2:**
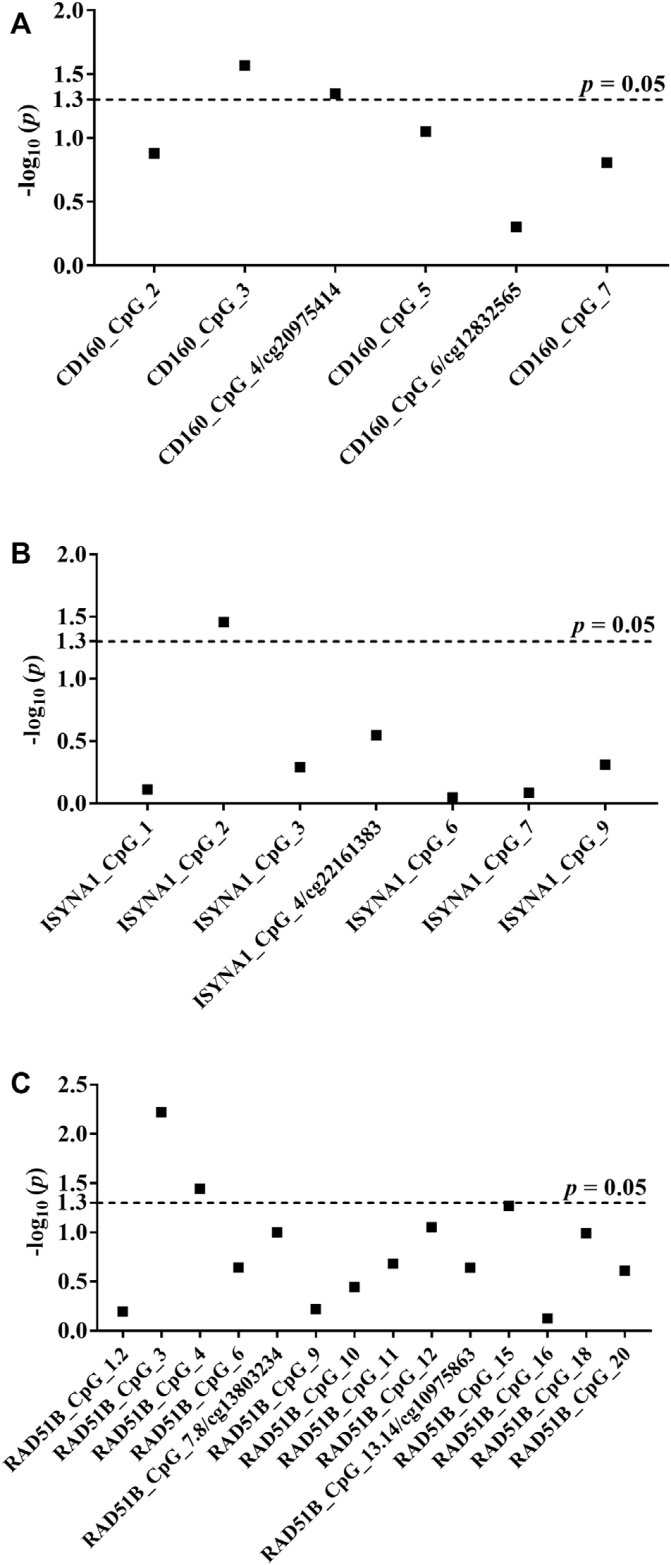
Association between hypomethylation of *CD160*, *ISYNA1*, and *RAD51B* and sporadic BC combining validation I and validation II. The *p* values of all measureable CpG loci were calculated by logistic regression adjusted for age and different batches for the measurements, and all the *p* values were transformed by −log_10_. The dotted lines indicate the thresholds of *p* values of 0.05. **(A)** Association between hypomethylation of *CD160* and sporadic BC combining validation I and validation II. **(B)** Association between hypomethylation of *ISYNA1* and sporadic BC combining validation I and validation II. **(C)** Association between hypomethylation of *RAD51B* and sporadic BC combining validation I and validation II.

After exclusion criteria were applied, six genes (*GBA*, *ATG10*, *TRIM27*, *CD160*, *ISYNA1*, *RAD51B*) were selected for validating the associations between DNA methylation and BC with MassARRAY EpiTyper assays in validation I (48 sporadic BC cases and 48 matched cancer-free female controls) (Supplementary Table S1). Among them, three genes (*CD160*, *ISYNA1*, *RAD51B*) showed methylation differences between BC cases and controls (Supplementary Table S1). Thus, these three genes were further validated in 224 sporadic BC cases and 224 matched female controls (validation II). Combining two validation studies with a total of 272 sporadic BC cases and 272 cancer-free female controls, we identified the blood-based hypomethylation in *CD160*, *ISYNA1*, and *RAD51B* as risk factors for BC in the Chinese population (CD160_CpG_3, CD160_cg20975414, ISYNA1_CpG_2, RAD51B_CpG_3 and RAD51B_CpG_4; odds ratios (ORs) per -10% methylation ranging from 1.08 to 1.67, *p <* 0.05 for all, by logistic regression adjusting for age and batch effects, Figure 2, Table 1).

**TABLE 1:** Association between hypomethylation of *CD160*, *ISYNA1*, and *RAD51B* and sporadic BC combining validation I and validation II.

CpG sites	Controls (*n* = 272)	BC cases (*n* = 272)	OR (95% CI)*	*p-*value*
	Median (IQR)	Median (IQR)	per -10% Methylation	
CD160_CpG_2	0.95 (0.85–0.98)	0.96 (0.85–0.99)	1.10 (0.97–1.24)	0.132
CD160_CpG_3	0.88 (0.66–1.00)	0.86 (0.59–1.00)	1.09 (1.01–1.17)	**0.027**
CD160_CpG_4/cg20975414	0.47 (0.30–0.66)	0.43 (0.24–0.65)	1.08 (1.00–1.16)	**0.045**
CD160_CpG_5	0.70 (0.54–0.86)	0.67 (0.46–0.84)	1.06 (0.99–1.14)	0.089
CD160_CpG_6/cg12832565	0.48 (0.34–0.62)	0.48 (0.28–0.65)	1.03 (0.95–1.11)	0.498
CD160_CpG_7	0.68 (0.56–0.85)	0.71 (0.54–0.83)	1.06 (0.98–1.16)	0.156
ISYNA1_CpG_1	0.85 (0.80–0.89)	0.85 (0.80–0.90)	1.03 (0.85–1.25)	0.773
ISYNA1_CpG_2	0.69 (0.60–0.77)	0.66 (0.57–0.77)	1.11 (1.01–1.23)	**0.035**
ISYNA1_CpG_3	0.87 (0.82–0.92)	0.87 (0.81–0.93)	1.07 (0.87–1.32)	0.511
ISYNA1_CpG_4/cg22161383	0.85 (0.80–0.91)	0.88 (0.81–0.93)	0.91 (0.77–1.08)	0.284
ISYNA1_CpG_6	0.34 (0.26–0.42)	0.33 (0.24–0.42)	0.99 (0.88–1.12)	0.896
ISYNA1_CpG_7	0.56 (0.48–0.64)	0.56 (0.47–0.65)	0.99 (0.87–1.12)	0.825
ISYNA1_CpG_9	0.55 (0.47–0.63)	0.56 (0.46–0.64)	0.96 (0.85–1.08)	0.488
RAD51B_CpG_1.2	0.65 (0.61–0.69)	0.65 (0.61–0.68)	0.92 (0.67–1.28)	0.639
RAD51B_CpG_3	0.97 (0.94–0.99)	0.96 (0.92–0.99)	1.67 (1.16–2.40)	**0.006**
RAD51B_CpG_4	0.68 (0.56–0.83)	0.67 (0.54–0.78)	1.10 (1.01–1.19)	**0.036**
RAD51B_CpG_6	0.64 (0.53–0.75)	0.65 (0.55–0.76)	0.94 (0.85–1.04)	0.228
RAD51B_CpG_7.8/cg13803234	0.83 (0.74–0.98)	0.85 (0.75–0.98)	0.90 (0.80–1.02)	0.100
RAD51B_CpG_9	0.57 (0.48–0.65)	0.58 (0.48–0.65)	0.97 (0.86–1.09)	0.602
RAD51B_CpG_10	0.36 (0.29–0.45)	0.38 (0.27–0.48)	0.95 (0.85–1.06)	0.359
RAD51B_CpG_11	0.31 (0.25–0.38)	0.32 (0.24–0.39)	0.91 (0.79–1.05)	0.208
RAD51B_CpG_12	0.34 (0.27–0.43)	0.36 (0.28–0.46)	0.89 (0.79–1.02)	0.089
RAD51B_CpG_13.14/cg10975863	0.45 (0.38–0.55)	0.48 (0.37–0.59)	0.93 (0.83–1.05)	0.229
RAD51B_CpG_15	0.32 (0.24–0.40)	0.34 (0.25–0.42)	0.89 (0.78–1.00)	0.054
RAD51B_CpG_16	0.27 (0.19–0.36)	0.25 (0.16–0.37)	1.02 (0.91–1.13)	0.748
RAD51B_CpG_18	0.47 (0.37–0.58)	0.50 (0.38–0.61)	0.92 (0.83–1.02)	0.102
RAD51B_CpG_20	0.64 (0.53–0.75)	0.65 (0.55–0.76)	0.94 (0.85–1.04)	0.245

*Logistic regression adjusted for age and different batches for the measurements.

Bold values indicated *p* < 0.05.

### Combination Analyses of the Association Between Blood-Based Methylation of *CD160*, *ISYNA1* and *RAD51B* and Breast Cancer Stratified by Age

Since age has impact on DNA methylation patterns (Horvath et al., 2012), we next evaluated the relationship between the methylation levels of *CD160*, *ISYNA1* and *RAD51B* and age in 272 sporadic BC cases and 272 cancer-free female controls combining validation I and validation II. As shown in Supplementary Table S2, the methylation levels of CD160_CpG_2, CD160_CpG_3 and CD160_CpG_5 were inversely correlated with age in controls (Spearman rho = −0.191, −0.150 and −0.146, respectively), whereas methylation levels of CD160_CpG_3 were inversely correlated with age in BC cases (Spearman rho = −0.240). In ISYNA1, only CpG_2 showed significantly positive correlation with age in BC cases (Spearman rho = 0.211). In RAD51B, cg13803234 showed positive correlation with age both in controls and in BC cases (Spearman rho = 0.224 and 0.195, respectively, Supplementary Table S2).

**FIGURE 3:**
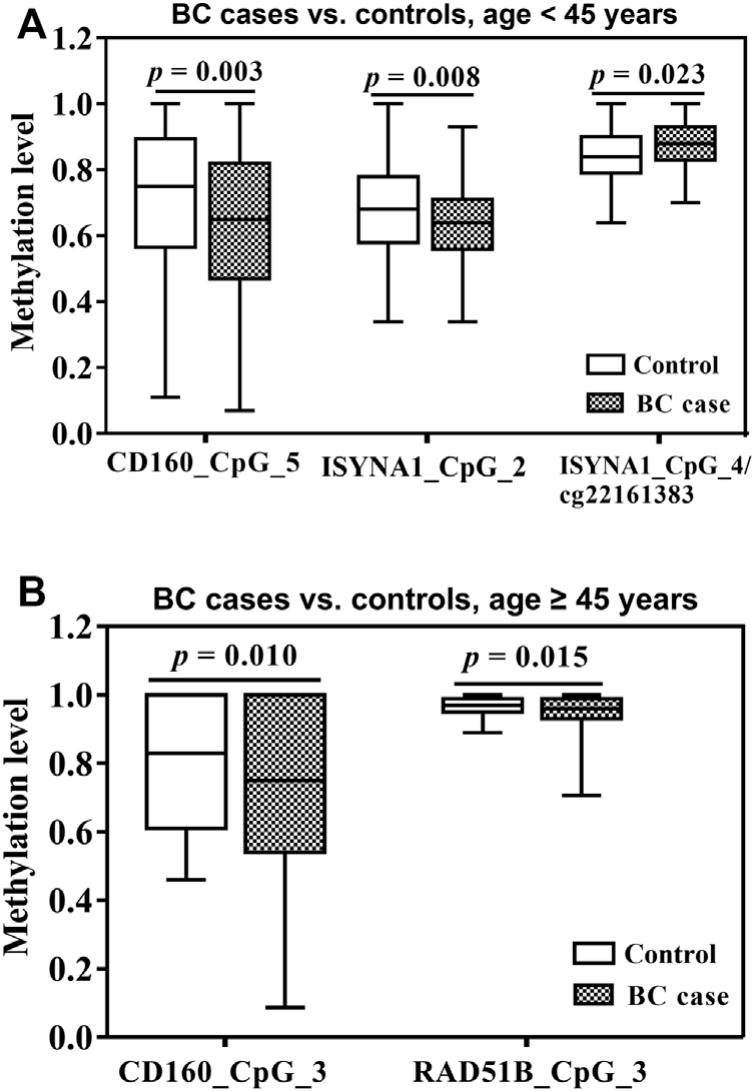
Association between blood-based methylation of *CD160*, *ISYNA1*, and *RAD51B* and BC stratified by age (45 years). The box plots show the distribution of DNA methylation levels in BC cases and controls. The *p* values were calculated by logistic regression adjusted for age and different batches for the measurements. **(A)** Association between blood-based methylation of *CD160*, *ISYNA1*, and *RAD51B* and BC in subjects <45 years old combining validation I and validation II **(B)** Association between blood-based methylation of CD160, ISYNA1, and RAD51B and BC in subjects ≥45 years old combining validation I and validation II.

Since *CD160*, *ISYNA1* and *RAD51B* showed differential age-related methylation patterns in controls and BC cases, we further stratified the subjects by 45 years old according to the median age combining two validation studies. In women younger than 45 years, CD160_CpG_5 and ISYNA1_CpG_2 showed significantly lower methylation levels in the BC cases than in the controls (ORs per -10% methylation = 1.21 and 1.26 respectively, *p* < 0.008 for both by logistic regression adjusted for age and batch effects, Figure 3A, Table 2), whereas ISYNA1_CpG_4 was hypermethylated in BC cases (OR per -10% methylation = 0.72, *p* = 0.023 by logistic regression, Figure 3A, Table 2). In the group ≥45 years, hypomethylation of CD160_CpG_3 and RAD51B_CpG_3 were significantly associated with increased risk of BC (ORs per -10% methylation = 1.16 and 2.31 respectively, *p* < 0.015 for both by logistic regression adjusted for age and batch effects, Figure 3B, Table 3).

**TABLE 2:** Association between blood-based methylation of *CD160*, *ISYNA1*, and *RAD51B* and BC in subjects younger than 45 years old combining validation I and validation II.

CpG sites	Controls (*n* = 131)	BC cases (*n* = 116)	OR (95% CI)*	*p-*value*
	Median (IQR)	Median (IQR)	per-10% Methylation	
CD160_CpG_2	0.96 (0.89–0.99)	0.97 (0.86–0.99)	1.14 (0.92–1.40)	0.244
CD160_CpG_3	0.99 (0.68–1.00)	0.96 (0.71–1.00)	1.03 (0.91–1.16)	0.631
CD160_CpG_4/cg20975414	0.46 (0.29–0.70)	0.39 (0.23–0.56)	1.10 (0.98–1.23)	0.122
CD160_CpG_5	0.75 (0.57–0.90)	0.65 (0.47–0.82)	1.21 (1.07–1.37)	**0.003**
CD160_CpG_6/cg12832565	0.51 (0.36–0.67)	0.51 (0.31–0.67)	1.08 (0.94–1.24)	0.257
CD160_CpG_7	0.68 (0.57–0.86)	0.74 (0.57–0.84)	1.08 (0.93–1.25)	0.345
ISYNA1_CpG_1	0.86 (0.81–0.89)	0.86 (0.81–0.90)	0.99 (0.72–1.35)	0.948
ISYNA1_CpG_2	0.68 (0.58–0.78)	0.64 (0.56–0.71)	1.26 (1.06–1.49)	**0.008**
ISYNA1_CpG_3	0.87 (0.82–0.92)	0.86 (0.81–0.93)	1.10 (0.79–1.54)	0.570
ISYNA1_CpG_4/cg22161383	0.84 (0.79–0.90)	0.88 (0.83–0.93)	0.72 (0.55–0.96)	**0.023**
ISYNA1_CpG_6	0.33 (0.27–0.42)	0.34 (0.26–0.44)	0.90 (0.72–1.13)	0.360
ISYNA1_CpG_7	0.57 (0.48–0.62)	0.56 (0.46–0.64)	1.01 (0.81–1.26)	0.930
ISYNA1_CpG_9	0.57 (0.49–0.63)	0.56 (0.45–0.62)	1.08 (0.87–1.35)	0.491
RAD51B_CpG_1.2	0.64 (0.60–0.68)	0.64 (0.61–0.68)	0.66 (0.36–1.22)	0.185
RAD51B_CpG_3	0.96 (0.92–0.99)	0.96 (0.92–0.99)	1.31 (0.75–2.28)	0.342
RAD51B_CpG_4	0.68 (0.57–0.83)	0.68 (0.59–0.77)	1.08 (0.93–1.26)	0.312
RAD51B_CpG_6	0.62 (0.51–0.72)	0.64 (0.55–0.74)	0.93 (0.78–1.11)	0.434
RAD51B_CpG_7.8/cg13803234	0.79 (0.71–0.89)	0.83 (0.73–0.90)	0.91 (0.73–1.14)	0.419
RAD51B_CpG_9	0.56 (0.47–0.62)	0.57 (0.49–0.64)	0.85 (0.68–1.07)	0.168
RAD51B_CpG_10	0.34 (0.29–0.43)	0.38 (0.26–0.48)	0.94 (0.77–1.15)	0.528
RAD51B_CpG_11	0.30 (0.25–0.37)	0.31 (0.23–0.38)	0.85 (0.66–1.09)	0.195
RAD51B_CpG_12	0.33 (0.27–0.42)	0.35 (0.27–0.45)	0.91 (0.72–1.15)	0.415
RAD51B_CpG_13.14/cg10975863	0.44 (0.38–0.53)	0.47 (0.37–0.57)	0.81 (0.65–1.01)	0.057
RAD51B_CpG_15	0.31 (0.24–0.39)	0.34 (0.25–0.42)	0.84 (0.67–1.04)	0.117
RAD51B_CpG_16	0.27 (0.20–0.36)	0.26 (0.19–0.40)	0.97 (0.81–1.17)	0.782
RAD51B_CpG_18	0.45 (0.37–0.54)	0.47 (0.37–0.58)	0.84 (0.69–1.02)	0.081
RAD51B_CpG_20	0.62 (0.51–0.72)	0.64 (0.55–0.74)	0.93 (0.78–1.11)	0.434

*Logistic regression adjusted for age and different batches for the measurements.

Bold values indicated *p* < 0.05.

**TABLE 3:** Association between blood-based methylation of *CD160*, *ISYNA1*, and *RAD51B* and BC in subjects older than or equal to 45 years old combining validation I and validation II.

CpG sites	Controls (*n* = 141)	BC cases (*n* = 156)	OR (95% CI)*	*p-*value*
	Median (IQR)	Median (IQR)	per-10% Methylation	
CD160_CpG_2	0.92 (0.81–0.98)	0.95 (0.83–0.98)	1.12 (0.93–1.35)	0.215
CD160_CpG_3	0.83 (0.61–1.00)	0.75 (0.54–1.00)	1.16 (1.04–1.30)	**0.010**
CD160_CpG_4/cg20975414	0.49 (0.32–0.63)	0.46 (0.24–0.69)	1.08 (0.96–1.21)	0.207
CD160_CpG_5	0.65 (0.48–0.82)	0.70 (0.46–0.86)	1.01 (0.91–1.13)	0.864
CD160_CpG_6/cg12832565	0.43 (0.31–0.59)	0.46 (0.27–0.64)	0.97 (0.87–1.09)	0.654
CD160_CpG_7	0.68 (0.55–0.83)	0.70 (0.50–0.82)	1.08 (0.95–1.22)	0.247
ISYNA1_CpG_1	0.84 (0.79–0.89)	0.85 (0.80–0.90)	1.05 (0.78–1.40)	0.756
ISYNA1_CpG_2	0.69 (0.61–0.77)	0.68 (0.58–0.81)	1.03 (0.89–1.19)	0.705
ISYNA1_CpG_3	0.87 (0.81–0.92)	0.88 (0.82–0.92)	1.00 (0.74–1.36)	0.986
ISYNA1_CpG_4/cg22161383	0.86 (0.81–0.91)	0.87 (0.78–0.93)	1.04 (0.80–1.35)	0.771
ISYNA1_CpG_6	0.34 (0.25–0.42)	0.33 (0.22–0.40)	1.05 (0.88–1.25)	0.628
ISYNA1_CpG_7	0.55 (0.48–0.64)	0.56 (0.48–0.66)	0.97 (0.81–1.18)	0.784
ISYNA1_CpG_9	0.54 (0.44–0.62)	0.56 (0.47–0.66)	0.89 (0.75–1.05)	0.164
RAD51B_CpG_1.2	0.66 (0.62–0.70)	0.65 (0.61–0.69)	0.98 (0.62–1.54)	0.919
RAD51B_CpG_3	0.97 (0.95–0.99)	0.96 (0.93–0.99)	2.31 (1.18–4.53)	**0.015**
RAD51B_CpG_4	0.69 (0.55–0.82)	0.65 (0.51–0.79)	1.12 (0.99–1.26)	0.068
RAD51B_CpG_6	0.66 (0.55–0.76)	0.67 (0.56–0.78)	0.97 (0.83–1.13)	0.701
RAD51B_CpG_7.8/cg13803234	0.88 (0.76–1.00)	0.87 (0.77–1.00)	0.91 (0.77–1.08)	0.275
RAD51B_CpG_9	0.58 (0.50–0.67)	0.58 (0.48–0.67)	1.08 (0.90–1.28)	0.409
RAD51B_CpG_10	0.38 (0.29–0.47)	0.39 (0.28–0.48)	0.96 (0.82–1.11)	0.564
RAD51B_CpG_11	0.32 (0.25–0.40)	0.32 (0.25–0.40)	0.94 (0.77–1.14)	0.509
RAD51B_CpG_12	0.36 (0.26–0.44)	0.37 (0.29–0.47)	0.93 (0.78–1.10)	0.401
RAD51B_CpG_13.14/cg10975863	0.48 (0.37–0.59)	0.48 (0.39–0.59)	0.99 (0.85–1.16)	0.940
RAD51B_CpG_15	0.34 (0.25–0.41)	0.33 (0.26–0.43)	0.93 (0.79–1.10)	0.384
RAD51B_CpG_16	0.27 (0.18–0.37)	0.25 (0.15–0.36)	1.04 (0.89–1.21)	0.625
RAD51B_CpG_18	0.49 (0.37–0.62)	0.50 (0.39–0.63)	0.96 (0.84–1.10)	0.602
RAD51B_CpG_20	0.66 (0.55–0.77)	0.67 (0.56–0.78)	0.98 (0.84–1.14)	0.780

*Logistic regression adjusted for age and different batches for the measurements.

Bold values indicated *p* < 0.05.

### Combination Analyses of the Correlation Between Altered Methylation in *CD160*, *ISYNA1* and *RAD51B* and Clinical Characteristics of Breast Cancer

Next, the relationship between *CD160, ISYNA1* and *RAD51B* methylation and the clinical characteristics of 272 sporadic BC cases was investigated. Lower methylation of CD160_CpG_5 and CD160_CpG_7 were observed in HER2-negative BC tumors and in triple negative tumors, respectively (*p* < 0.05, Table 4). The BC cases with advanced stages (stage II and stage III), larger tumor size (T2&T3&T4), and more lymph node involvement (pN1&pN2&pN3) had lower ISYNA1_cg22161383 methylation than the patients with stage 0&I tumor, smaller tumor (T0&T1), and no lymph node involvement (*p* < 0.019, Table 5). In RAD51B, CpG_6 and CpG_20 showed lower methylation levels in BC patients with more lymph node involvement (pN1&pN2&pN3) (*p* = 0.001 for both; Table 6). Additionally, hypomethylation of RAD51B_CpG_1.2, RAD51B_CpG_3 and RAD51B_cg13803234 was correlated with ER-negative status, hypomethylation of RAD51B_CpG_1.2 was correlated with PR-negative status, and hypomethylation of RAD51B_CpG_1.2, RAD51B_CpG_3, RAD51B_cg13803234, RAD51B_CpG_10, RAD51B_CpG_11 and RAD51B_CpG_18 was correlated with triple-negative BC (*p* < 0.05, Table 6). Other CpG sites in *CD160, ISYNA1* and *RAD51B* showed no or borderline correlations with clinical characteristics of BC.

**TABLE 4:** Correlation between *CD160* methylation and the clinical characteristics of sporadic BC cases combining validation I and validation II.

Characteristics	Group (*n*)	Median of methylation levels					
		CD160_CpG_2	CD160_CpG_3	CD160_CpG_4/cg20975414	CD160_CpG_5	CD160_CpG_6/cg12832565	CD160_CpG_7
Tumor stage	Stage 0&Ⅰ (116)	0.96	0.85	0.42	0.71	0.49	0.72
	Stage Ⅱ&Ⅲ (131)	0.96	0.87	0.47	0.63	0.47	0.71
	*p-*value*	0.853	0.849	0.705	0.257	0.759	0.560
Tumor size	T0&1 (158)	0.96	0.85	0.43	0.66	0.49	0.71
	T2&3&4 (91)	0.97	0.88	0.47	0.66	0.44	0.71
	*p-*value*	0.590	0.539	0.812	0.863	0.595	0.681
Lymph node involvement	N0 (144)	0.96	0.85	0.46	0.70	0.49	0.72
	N1&2&3 (103)	0.96	0.88	0.42	0.60	0.46	0.71
	*p-*value*	0.777	0.726	0.498	0.146	0.571	0.951
Ki67	Ki67 ≤ 20% (95)	0.95	0.81	0.41	0.66	0.47	0.72
	Ki67 > 20% (154)	0.96	0.88	0.44	0.68	0.49	0.71
	*p-*value*	0.206	0.440	0.965	0.575	0.436	0.515
ER	ER negative (52)	0.95	0.87	0.43	0.74	0.50	0.71
	ER positive (200)	0.96	0.86	0.44	0.66	0.47	0.71
	*p-*value*	0.593	0.707	0.714	0.349	0.238	0.566
PR	PR negative (68)	0.97	0.85	0.43	0.74	0.52	0.70
	PR positive (184)	0.96	0.86	0.44	0.64	0.47	0.72
	*p-*value*	0.548	0.307	0.399	0.182	0.056	0.437
HER2	HER2 negative (191)	0.95	0.86	0.43	0.64	0.48	0.71
	HER2 positive (62)	0.97	0.87	0.46	0.75	0.49	0.72
	*p-*value*	0.208	0.26	0.527	**0.048**	0.996	0.3
Triple-negative	Triple-negative (32)	0.93	0.82	0.41	0.68	0.51	0.60
	Non-triple-negative (220)	0.96	0.87	0.44	0.66	0.48	0.72
	*p-*value*	0.110	0.544	0.254	0.823	0.530	**0.031**

*Mann-Whitney U test.

Bold values indicated *p* < 0.05.

**TABLE 5:** Correlation between *ISYNA1* methylation and the clinical characteristics of sporadic BC cases combining validation I and validation II.

Characteristics	Group (*n*)	Median of methylation levels						
		ISYNA1_CpG_1	ISYNA1_CpG_2	ISYNA1_CpG_3	ISYNA1_CpG_4/cg22161383	ISYNA1_CpG_6	ISYNA1_CpG_7	ISYNA1_CpG_9
Tumor stage	Stage 0&Ⅰ (116)	0.85	0.67	0.87	0.90	0.32	0.57	0.53
	Stage Ⅱ&Ⅲ (131)	0.86	0.66	0.87	0.86	0.34	0.56	0.57
	*p-*value*	0.147	0.235	0.953	**4.12E−04**	0.531	0.965	**0.047**
Tumor size	T0&1 (158)	0.85	0.66	0.87	0.89	0.33	0.57	0.55
	T2&3&4 (91)	0.86	0.67	0.87	0.85	0.34	0.54	0.56
	*p-*value*	0.353	0.868	0.852	**0.003**	0.868	0.481	0.188
Lymph node involvement	N0 (144)	0.85	0.67	0.87	0.89	0.33	0.56	0.55
	N1&2&3 (103)	0.86	0.66	0.88	0.86	0.34	0.58	0.57
	*p-*value*	0.119	0.106	0.461	**0.019**	0.447	0.287	0.155
Ki67	Ki67 ≤ 20% (95)	0.84	0.66	0.87	0.88	0.33	0.56	0.54
	Ki67 > 20% (154)	0.86	0.67	0.87	0.87	0.33	0.56	0.57
	*p-*value*	0.120	0.883	0.544	0.477	0.872	0.780	0.508
ER	ER negative (52)	0.86	0.68	0.88	0.87	0.31	0.58	0.58
	ER positive (200)	0.85	0.66	0.87	0.88	0.33	0.56	0.55
	*p-*value*	0.385	0.347	0.793	0.592	0.875	0.770	0.316
PR	PR negative (68)	0.86	0.68	0.88	0.87	0.32	0.57	0.57
	PR positive (184)	0.86	0.66	0.87	0.88	0.33	0.56	0.55
	*p-*value*	0.605	0.173	0.749	0.958	0.995	0.725	0.445
HER2	HER2 negative (191)	0.86	0.66	0.87	0.88	0.33	0.56	0.55
	HER2 positive (62)	0.85	0.68	0.89	0.88	0.33	0.58	0.56
	*p-*value*	0.877	0.402	0.129	0.957	0.859	0.412	0.784
Triple-negative	Triple-negative (32)	0.89	0.67	0.86	0.87	0.31	0.59	0.58
	Non-triple-negative (220)	0.85	0.67	0.87	0.88	0.33	0.56	0.55
	*p-*value*	0.056	0.958	0.685	0.463	0.873	0.975	0.274

*Mann-Whitney U test.

Bold values indicated *p* < 0.05.

**TABLE 6:** Correlation between *RAD51B* methylation and the clinical characteristics of sporadic BC cases combining validation I and validation II.

Characteristics	Group (N)	Median of methylation levels													
		RAD51B_CpG_1.2	RAD51B_CpG_3	RAD51B_CpG_4	RAD51B_CpG_6	RAD51B_CpG_7.8/<cg13803234	RAD51B_CpG_9	RAD51B_CpG_10	RAD51B_CpG_11	RAD51B_CpG_12	RAD51B_CpG_13.14/cg10975863	RAD51B_CpG_15	RAD51B_CpG_16	RAD51B_CpG_18	RAD51B_CpG_20
Tumor stage	Stage 0&Ⅰ (116)	0.65	0.96	0.68	0.64	0.855	0.575	0.37	0.32	0.365	0.48	0.34	0.25	0.50	0.64
	Stage Ⅱ&Ⅲ (131)	0.65	0.96	0.67	0.65	0.84	0.58	0.39	0.31	0.36	0.47	0.34	0.26	0.49	0.65
	*p-*value*	0.377	0.475	0.231	0.275	0.225	0.918	0.803	0.497	0.943	0.722	0.986	0.742	0.432	0.275
Tumor size	T0&1 (158)	0.65	0.96	0.66	0.64	0.85	0.57	0.37	0.32	0.35	0.47	0.34	0.25	0.50	0.64
	T2&3&4 (89)	0.65	0.96	0.68	0.67	0.84	0.58	0.40	0.32	0.37	0.48	0.34	0.27	0.50	0.67
	*p-*value*	0.620	0.513	0.349	0.296	0.413	0.227	0.204	0.382	0.192	0.163	0.317	0.804	0.992	0.296
Lymph node involvement	N0 (144)	0.65	0.96	0.68	0.69	0.86	0.58	0.39	0.32	0.37	0.48	0.35	0.26	0.51	0.69
	N1&2&3 (103)	0.64	0.96	0.64	0.62	0.84	0.56	0.37	0.30	0.35	0.47	0.32	0.25	0.47	0.62
	*p-*value*	0.098	0.931	0.076	**0.001**	0.053	0.420	0.325	0.127	0.251	0.230	0.053	0.640	0.088	**0.001**
Ki67	Ki67 ≤ 20% (94)	0.65	0.96	0.68	0.63	0.86	0.57	0.39	0.32	0.35	0.47	0.35	0.28	0.50	0.63
	Ki67 > 20% (154)	0.65	0.96	0.67	0.67	0.84	0.58	0.38	0.32	0.37	0.48	0.33	0.25	0.49	0.67
	*p-*value*	0.915	0.150	0.822	0.313	0.882	0.757	0.804	0.783	0.817	0.993	0.262	0.664	0.750	0.313
ER	Negative (52)	0.64	0.96	0.68	0.64	0.84	0.57	0.38	0.31	0.36	0.47	0.34	0.26	0.49	0.64
	Positive (200)	0.66	0.97	0.67	0.67	0.88	0.60	0.40	0.34	0.38	0.50	0.34	0.25	0.51	0.67
	*p-*value*	**0.038**	**0.043**	0.406	0.375	**0.028**	0.369	0.337	0.083	0.436	0.324	0.963	0.569	0.183	0.375
PR	Negative (68)	0.64	0.96	0.68	0.64	0.84	0.57	0.37	0.31	0.35	0.46	0.34	0.26	0.49	0.64
	Positive (184)	0.66	0.96	0.67	0.67	0.86	0.59	0.40	0.33	0.38	0.53	0.34	0.25	0.52	0.67
	*p-*value*	**0.035**	0.086	0.497	0.219	0.111	0.176	0.200	0.074	0.247	0.056	0.561	0.652	0.079	0.219
HER2	Negative (190)	0.65	0.96	0.67	0.65	0.84	0.58	0.39	0.32	0.36	0.47	0.34	0.27	0.50	0.65
	Positive (62)	0.65	0.96	0.68	0.65	0.88	0.57	0.37	0.32	0.36	0.49	0.32	0.25	0.50	0.65
	*p-*value*	0.756	0.582	0.260	0.659	0.191	0.946	0.582	0.974	0.893	0.585	0.824	0.480	0.589	0.659
Triple-negative	Triple-negative (32)	0.64	0.96	0.68	0.64	0.84	0.57	0.37	0.31	0.35	0.47	0.33	0.25	0.49	0.64
	Non-triple-negative (220)	0.67	0.98	0.65	0.74	0.92	0.61	0.46	0.38	0.41	0.54	0.39	0.26	0.58	0.74
	*p-*value*	**0.006**	**0.038**	0.391	0.083	**0.025**	0.059	**0.017**	**0.003**	0.076	0.067	0.195	0.306	**0.034**	0.083

*Mann-Whitney U test.

Bold values indicated *p* < 0.05.

## Discussion

Yang et al. (Yang et al., 2020) have reported BC-related methylation in peripheral blood in the European population. Here, we validated the associations in the Chinese population in two independent case-control studies with a total of 544 subjects. Our results supported the previous findings (Yang et al., 2020) that altered methylation of *CD160*, *ISYNA1* and *RAD51B* in the peripheral blood was associated with BC.

CD160, ISYNA1 and RAD51B have been involved in the development of various types of cancer. CD160 (cluster of differentiation 160), also known as natural killer cell receptor BY55, plays a role in human cancers such as chronic lymphocytic leukemia (CLL) (Bozorgmehr et al., 2021), colon cancer and melanoma (Chabot et al., 2011; Saleh et al., 2020), and pancreatic cancer (Liu et al., 2020). Bozorgmehr et al. (Bozorgmehr et al., 2021) found that CD160 was upregulated in patients with CLL and its expression was associated with an exhausted T cell phenotype, implicating an important role of CD160 in T cell exhaustion in patients with CLL. Chabot et al. (Chabot et al., 2011) found overexpression of CD160 on endothelial cells of newly formed blood vessels in human colon cancer and mouse B16 melanoma, but not in vessels of healthy tissues, suggesting its potential roles in the development and progression of cancer. Liu et al. (Liu et al., 2020) observed that tumor-infiltrating CD8^+^ T cells were significantly enriched with the CD160^+^ subset in pancreatic cancer patients, and patients with higher frequencies of tumor CD160^+^CD8^+^ T cells presented lower survival. Farren and colleagues have shown that the CD160 is overexpressed in malignant B cells, but not in healthy B cells, indicating CD160 as a tumor-specific marker of malignant B lymphocytes (Farren et al., 2011). ISYNA1 (inositol-3-phosphate synthase1) is a rate-limiting enzyme that catalyzes the biosynthesis of inositol, which regulates glycolipid metabolism, neurotropic effects and tumor suppression (Croze and Soulage, 2013). Activated *p53* could regulate *ISYNA1* expression in the cells, and knockdown of *ISYNA1* caused resistance to adriamycin treatment, demonstrating the role of *ISYNA1* in *p53*-mediated growth suppression (Koguchi et al., 2016). Moreover, higher expression of *ISYNA1* is associated with gliomas and bladder carcinoma (Nagashima et al., 2018; Guo et al., 2019). RAD51B (RAD51 paralog B) is an important member of the RAD51 protein family, which are evolutionarily conserved and essential for DNA repair by homologous recombination (Suwaki et al., 2011). RAD51B plays a vital role in homologous recombinational repair of DNA double-strand breaks to maintain cell genomic stability and is a promising candidate oncogene and biomarker for cancer diagnosis and prognosis (Nagathihalli and Nagaraju, 2011; Terasawa et al., 2014; Cheng et al., 2016). Cheng et al. (Cheng et al., 2016) showed that the mRNA expression of *RAD51B* was significantly elevated in gastric cancer tissues, and patients with high level of *RAD51B* expression exhibited worse overall survival. Additionally, functional studies indicated that over-expression of *RAD51B* promoted the proliferation of gastric cancer cell, while *RAD51B* knockdown led to G1 arrest, suggesting that *RAD51B* may act as an oncogene during gastric cancer progression.

In our study, we observed significantly lower methylation of *CD160*, *ISYNA1* and *RAD51B* in blood DNA of BC patients than that of cancer-free controls in the Chinese population. In contrast, Yang et al. (Yang et al., 2020) have shown that DNA methylation levels of *CD160*, *ISYNA1* and *RAD51B* are positively correlated with BC risk in the European population. The differential DNA methylation patterns between ethnicities have been reported previously, including significant differences in global leukocyte DNA methylation by race/ethnicity (Zhang et al., 2011), differences in smoking-associated DNA methylation patterns in South Asians and Europeans (Elliott et al., 2014), as well as race-specific alterations in DNA methylation among African Americans and Caucasians (Chitrala et al., 2020). The major mechanism for the epigenetic related hereditary background is the differential genetic variations and frequencies in different populations. Genetic studies have identified ethnic differences in gene polymorphisms of *CD160* in autoimmune diseases and *RAD51B* in BC (Hua et al., 2012; Kurreeman et al., 2012; Zhu et al., 2016; Li et al., 2018; He et al., 2021). Although so far there are no studies about the variations of *ISYNA1* in different ethnic groups, the upstream genetic variations may modulate the regulation of *ISYNA1*. Indeed, the methylation/expression of *ISYNA1* is regulated by *P53* (Koguchi et al., 2016), and different mutations in *P53* gene have been reported in BC among different ethnic groups (Huo et al., 2017). On the other hand, environmental exposures and life styles may contribute to differences in DNA methylation as well (Delgado-Cruzata et al., 2015; Abdul et al., 2017; Nwanaji-Enwerem and Colicino, 2020; Wang et al., 2020). For example, the environmental carcinogen pollutions, such as compounds in combustion gases and in cigarette smoke generally cause global DNA hypomethylation (but hypermethylate the tumor suppressor genes), increasing the likelihood of cancers, including BC (Xue et al., 2011; Lee and Pausova, 2013; Goldvaser et al., 2017; Martin and Fry, 2018). Moreover, the life style habits including diets that are abundant in xenoestrogens and nutrition profile, consumption of inflammatory and carcinogen foods or anti-inflammatory and chemo-preventive foods, in particular, also influences global DNA methylation and is relevant to cancer risk (Glade, 1999; Johanning et al., 2002; Shaikh et al., 2019; Maugeri and Barchitta, 2020). Those DNA methylation alterations, once established, can persist in the absence of the initial environmental or life style factors. However, due to the limitation of hospital-based sample collection, the environmental factors and life style factors such as smoking habits and diets were unfortunately not available in this study. Further analyses including information of life styles and environmental factors in future studies with larger sample size are warranted. Taken together, genetic background and different life styles could be confounders for *CD160*, *ISYNA1* and *RAD51B* -associated BC risk in different ethnicities.

Mounting evidences have disclosed that the DNA methylation in human peripheral leukocytes could vary with age (Fuke et al., 2004; Jung et al., 2017). Thus, in our study, we evaluated the correlation between methylation of *CD160*, *ISYNA1* and *RAD51B* and age, and further compared the DNA methylation levels between BC cases and controls in different age groups. We observed a significant correlation between methylation levels of *CD160*, *ISYNA1* and *RAD51B* and age either in controls or in cases. Significantly lower methylation levels of *CD160*, *ISYNA1* and *RAD51B* in cases than controls were further found in women of different age groups. Our results suggested that age might be a confounder for the cancer associated aberrant methylation of *CD160*, *ISYNA1* and *RAD51B* in the blood. To better understand the role of age on the blood-based methylation changes, further mechanism studies of *CD160*, *ISYNA1* and *RAD51B* and aging is warranted in the future.

Our data revealed that DNA methylation was related to the clinical characteristics of BC, consistent with our previous studies (Lei et al., 2021; Yin et al., 2022). Here we found significantly lower methylation of *CD160*, *ISYNA1* and *RAD51B* were correlated with hormone receptor status, increased breast tumor size, advanced tumor stage and more lymph node involvement. Differential expression of *CD160*, *ISYNA1* and *RAD51B* has been correlated to the clinical characteristics in various types of cancer. Yu et al. (Yu et al., 2021) observed elevated mRNA levels of *CD160* in diffuse large B cell lymphoma, especially in subtype I, which displayed poorer overall survival time and progression-free survival time than those in subtype II. Nagashima *et al.* (Nagashima et al., 2018) found significantly higher expression of *ISYNA1* in high-grade gliomas than in primary central nervous system lymphomas. Cheng et al. (Cheng et al., 2016) showed that *RAD51B* mRNA expression was significantly up-regulated in gastric cancer tissues and high level of RAD51B protein was correlated with advanced stage, aggressive differentiation and lymph node metastasis. However, so far there are no reports about the correlation between *CD160*, *ISYNA1* as well as *RAD51B* and clinical characteristics of BC, especially in the aspect of DNA methylation. Our findings suggested that aberrant methylation of *CD160*, *ISYNA1* and *RAD51B* in blood might be important predictors for the development of BC and could be prognosis biomarkers for BC. Unfortunately, the samples of fresh blood or RNA are not available in this study. Whether the altered methylation of *CD160*, *ISYNA1* and *RAD51B* could modulate the gene expression and biological function requires further investigations in future. DNA methyltransferases (DNMTs) are responsible for the establishment and maintenance of DNA methylation (Edwards et al., 2017; Lyko, 2018). Lysine demethylases (KDMs) are responsible for the demethylation of histone H3 and non-histone substrates, and have been implicated in diverse genomic processes, such as epigenetic gene regulation, DNA damage response, DNA replication, regulation of heterochromatin structure and maintenance of global DNA methylation (Dimitrova et al., 2015; Arifuzzaman et al., 2020). DNMTs and KDMs (like KDM4 and KDM5) are often deregulated and play important roles in malignant tumors (Plch et al., 2019; Hoang and Rui, 2020; Lee et al., 2020; Sterling et al., 2020). Therefore, it would be interesting to investigate the expression status of key DNMTs and KDMs, and analyze their relationship with the altered methylation levels of *CD160*, *ISYNA1* and *RAD51B* in the peripheral blood of BC cases and controls, which could further validate the results presented here. In addition, methylation of cytosine in CpG dinucleotides and histone lysine and arginine residues is a chromatin modification that regulates genome integrity, replication, and accessibility (Rose and Klose, 2014; Li et al., 2021). Genome-wide profiling of CpG methylation revealed a strong correlation between DNA methylation and histone methylation, including a positive correlation with histone H3K9 methylation and a negative correlation with H3K4 methylation (Meissner et al., 2008). A meta-analysis of whole-genome bisulfite sequencing and ChIP-seq datasets from 35 human cell types also showed that CpG methylation is negatively correlated with H3K4 and H3K27 methylation and positively correlated with H3K9 and H3K36 methylation (Fu et al., 2020). In addition, several evidences have disclosed an intimate interaction between DNA and histone methylation in the development of human diseases. Hypermethylation in promoter CpG islands in cancers are marked by H3K27me3 in embryonic or tissue stem/progenitor cells (Ohm et al., 2007; Schlesinger et al., 2007; Widschwendter et al., 2007). Dunican et al. (Dunican et al., 2020) showed that the ratio of H3K27me3 to H3K4me3 at bivalent promoters can predict the likelihood of cancer-associated DNA hypermethylation. Stoll et al. (Stoll et al., 2018) demonstrated that DNA methylation and histone methylation are involved in the function of vascular cells in response to environmental stresses. Therefore, the data relating to histone methylation status could help to interpret DNA methylation status of CpG sites, which are not available in the study and need further investigation in the future studies.

Moreover, due to the limitation of the case-control studies with relatively small samples, large-scale prospective studies are warranted to further validate our results and identify if such DNA methylation signatures could bear on the diagnosis and/or prognosis of patients with BC.

## Conclusion

This study provided further evidence for the association between altered methylation of *CD160*, *ISYNA1* and *RAD51B* in blood and BC. In addition, we suggested the influence of genetic background, life style, age, stage and receptor status of tumor as confounders for the DNA methylation. Notably, we highlighted that the epigenetic biomarkers in one ethnic group warrant population-based validation before its application in another ethnic group.

## Data Availability

The original contributions presented in the study are included in the article/Supplementary Materials, further inquiries can be directed to the corresponding author.
