# Supplementary material for: The Association Between Breast Cancer and Blood-Based Methylation of CD160, ISYNA1 and RAD51B in the Chinese Population
Source: Front Genet. 2022 Jun 9;13:927519. doi: 10.3389/fgene.2022.927519 (PMC9261985; doi:10.3389/fgene.2022.927519)
Supplement: Supplementary file 1 [file DataSheet1.doc]

Supplementary Table 1. Validation of BC-associated DNA methylation markers in validation I

| CpG sites | Controls (n=48) | Cases (n=48) | OR (95%CI)* | *p*-value* |
| --- | --- | --- | --- | --- |
|  | Median (IQR) | Median (IQR) | per-10% methylation |  |
| GBA_CpG_1 | 0.05 (0.04-0.06) | 0.05 (0.03-0.06) | 3.35 (0.29-39.21) | 0.336 |
| GBA_CpG_2 | 0.00 (0.00-0.02) | 0.01 (0.00-0.01) | 0.28 (0.01-7.89) | 0.455 |
| GBA_CpG_3 | 0.11 (0.07-0.16) | 0.10 (0.06-0.14) | 1.38 (0.78-2.43) | 0.271 |
| GBA_CpG_5/cg02834765 | 0.05 (0.00-0.08) | 0.02 (0.00-0.09) | 1.12 (0.66-1.90) | 0.681 |
| GBA_CpG_6 | 0.00 (0.00-0.01) | 0.00 (0.00-0.01) | 0.16 (0.00-16.63) | 0.440 |
| GBA_CpG_7 | 0.16 (0.15-0.19) | 0.17 (0.15-0.19) | 0.51 (0.11-2.46) | 0.402 |
| GBA_CpG_8 | 0.04 (0.03-0.04) | 0.04 (0.03-0.05) | 0.23 (0.02-3.28) | 0.280 |
| GBA_CpG_9 | 0.03 (0.02-0.03) | 0.03 (0.02-0.03) | 0.24 (0.00-55.03) | 0.608 |
| ATG10_CpG_1/cg25363080 | 0.19 (0.17-0.24) | 0.18 (0.13-0.25) | 1.07 (0.60-1.90) | 0.833 |
| ATG10_CpG_2 | 0.42 (0.33-0.51) | 0.38 (0.30-0.49) | 0.97 (0.77-1.23) | 0.820 |
| ATG10_CpG_3 | 0.61 (0.54-0.67) | 0.64 (0.54-0.71) | 0.92 (0.66-1.28) | 0.629 |
| ATG10_CpG_4 | 0.37 (0.31-0.47) | 0.37 (0.28-0.51) | 0.90 (0.67-1.19) | 0.446 |
| TRIM27_CpG_1.2 | 0.92 (0.88-­0.96) | 0.92 (0.89­-0.95) | 0.92 (0.02-4.76) | 0.967 |
| TRIM27_CpG_3/cg05216056 | 0.49 (0.37-­0.64) | 0.49 (0.36­-0.60) | 1.30 (0.32­-5.33) | 0.719 |
| TRIM27_CpG_4 | 1.00 (0.96­-1.00) | 1.00 (0.98-­1.00) | 0.10 (0.0­1-43.85) | 0.539 |
| TRIM27_CpG_5 | 0.66 (0.58­-0.79) | 0.67 (0.60-­0.77) | 1.90 (0.32­-11.46) | 0.483 |
| CD160_CpG_2 | 0.95 (0.82-0.99) | 0.95 (0.71- 0.98) | 1.29 (0.97-1.71) | 0.079 |
| CD160_CpG_3 | 0.86 (0.67-1.00) | 0.89 (0.63-1.00) | 1.01 (0.86-1.18) | 0.935 |
| CD160_CpG_4/cg20975414 | 0.40 (0.28-0.54) | 0.30 (0.20-0.52) | 1.12 (0.93-1.35) | 0.230 |
| CD160_CpG_5 | 0.67 (0.56-0.87) | 0.59 (0.35-0.77) | 1.21 (1.01-1.44) | **0.039** |
| CD160_CpG_6/cg12832565 | 0.45 (0.35-0.58) | 0.34 (0.16-0.62) | 1.22 (1.00-1.49) | **0.048** |
| CD160_CpG_7 | 0.64 (0.55-0.75) | 0.68 (0.36-0.82) | 1.10 (0.90-1.34) | 0.372 |
| ISYNA1_CpG_1 | 0.86 (0.83-0.88) | 0.86 (0.82-0.91) | 0.80 (0.41-1.55) | 0.507 |
| ISYNA1_CpG_2 | 0.65 (0.58-0.71) | 0.65 (0.59-0.71) | 0.97 (0.73-1.28) | 0.807 |
| ISYNA1_CpG_3 | 0.87 (0.83-0.91) | 0.84 (0.75-0.90) | 1.95 (1.10-3.46) | **0.022** |
| ISYNA1_CpG_4/cg22161383 | 0.86 (0.84-0.89) | 0.84 (0.76-0.89) | 1.98 (1.14-3.43) | **0.015** |
| ISYNA1_CpG_6 | 0.35 (0.30-0.39) | 0.34 (0.25-0.44) | 1.10 (0.74-1.65) | 0.645 |
| ISYNA1_CpG_7 | 0.57 (0.50-0.62) | 0.59 (0.47-0.68) | 0.91 (0.63-1.31) | 0.597 |
| ISYNA1_CpG_9 | 0.57 (0.51-0.63) | 0.52 (0.40-0.59) | 1.55 (1.05-2.29) | **0.026** |
| RAD51B_CpG_1.2 | 0.62 (0.59-0.65) | 0.62 (0.59-0.65) | 0.77 (0.32-1.85) | 0.562 |
| RAD51B_CpG_3 | 0.95 (0.92-0.99) | 0.96 (0.90-0.99) | 1.77 (0.71-4.37) | 0.218 |
| RAD51B_CpG_4 | 0.67 (0.57-0.85) | 0.63 (0.54-0.74) | 1.17 (0.95-1.45) | 0.143 |
| RAD51B_CpG_6 | 0.71 (0.63-0.76) | 0.67 (0.60-0.76) | 1.11 (0.85-1.46) | 0.438 |
| RAD51B_CpG_7.8/cg13803234 | 0.86 (0.79-0.98) | 0.86 (0.80-0.97) | 0.93 (0.68-1.28) | 0.663 |
| RAD51B_CpG_9 | 0.59 (0.53-0.65) | 0.58 (0.51-0.65) | 1.24 (0.83-1.87) | 0.295 |
| RAD51B_CpG_10 | 0.38 (0.33-0.48) | 0.39 (0.33-0.51) | 1.03 (0.71-1.49) | 0.873 |
| RAD51B_CpG_11 | 0.31 (0.27-0.40) | 0.31 (0.25-0.35) | 1.20 (0.74-1.94) | 0.458 |
| RAD51B_CpG_12 | 0.37 (0.32-0.45) | 0.38 (0.32-0.48) | 1.04 (0.68-1.59) | 0.843 |
| RAD51B_CpG_13.14/cg10975863 | 0.50 (0.45-0.62) | 0.49 (0.42-0.60) | 1.28 (0.89-1.83) | 0.184 |
| RAD51B_CpG_15 | 0.37 (0.32-0.43) | 0.36 (0.29-0.46) | 1.09 (0.75-1.58) | 0.641 |
| RAD51B_CpG_16 | 0.35 (0.27-0.45) | 0.24 (0.18-0.38) | 1.52 (1.13-2.03) | **0.005** |
| RAD51B_CpG_18 | 0.53 (0.46-0.62) | 0.50 (0.40-0.58) | 1.31 (0.96-1.79) | 0.093 |
| RAD51B_CpG_20 | 0.71 (0.63-0.76) | 0.67 (0.60-0.76) | 1.11 (0.85-1.46) | 0.438 |

*Logistic regression adjusted for age; Bold values indicated *p* < 0.05.

Supplementary Table 2. Correlations between the methylation levels of *CD160, ISYNA1* and *RAD51B* and age combining validation I and validation II

| CpG sites | Age (272 controls) | |  | Age (272 BC cases) | |
| --- | --- | --- | --- | --- | --- |
|  | Spearman rho | *p-value* |  | Spearman rho | *p-value* |
| CD160_CpG_2 | -0.191 | **0.002** |  | -0.105 | 0.096 |
| CD160_CpG_3 | -0.150 | **0.016** |  | -0.240 | **1.16E-04** |
| CD160_CpG_4/cg20975414 | 0.023 | 0.718 |  | 0.037 | 0.553 |
| CD160_CpG_5 | -0.146 | **0.020** |  | 0.050 | 0.425 |
| CD160_CpG_6/cg12832565 | -0.043 | 0.491 |  | -0.054 | 0.390 |
| CD160_CpG_7 | 0.014 | 0.828 |  | -0.063 | 0.321 |
| ISYNA1_CpG_1 | -0.025 | 0.691 |  | -0.037 | 0.559 |
| ISYNA1_CpG_2 | 0.092 | 0.137 |  | 0.211 | **0.001** |
| ISYNA1_CpG_3 | -0.095 | 0.126 |  | 0.098 | 0.119 |
| ISYNA1_CpG_4/cg22161383 | 0.089 | 0.153 |  | 0.010 | 0.872 |
| ISYNA1_CpG_6 | -0.050 | 0.417 |  | -0.077 | 0.222 |
| ISYNA1_CpG_7 | -0.048 | 0.442 |  | 0.026 | 0.678 |
| ISYNA1_CpG_9 | -0.097 | 0.122 |  | 0.094 | 0.137 |
| RAD51B_CpG_1.2 | 0.100 | 0.107 |  | 0.097 | 0.121 |
| RAD51B_CpG_3 | 0.108 | 0.081 |  | 0.067 | 0.288 |
| RAD51B_CpG_4 | -0.019 | 0.755 |  | -0.112 | 0.074 |
| RAD51B_CpG_6 | 0.080 | 0.198 |  | 0.102 | 0.105 |
| RAD51B_CpG_7.8/cg13803234 | 0.224 | **2.6E-04** |  | 0.195 | **0.002** |
| RAD51B_CpG_9 | 0.076 | 0.222 |  | -0.004 | 0.944 |
| RAD51B_CpG_10 | 0.071 | 0.250 |  | 0.005 | 0.935 |
| RAD51B_CpG_11 | 0.025 | 0.686 |  | 0.024 | 0.701 |
| RAD51B_CpG_12 | 0.025 | 0.687 |  | 0.030 | 0.634 |
| RAD51B_CpG_13.14/cg10975863 | 0.036 | 0.560 |  | -0.019 | 0.758 |
| RAD51B_CpG_15 | 0.037 | 0.549 |  | -0.038 | 0.542 |
| RAD51B_CpG_16 | -0.052 | 0.404 |  | -0.089 | 0.155 |
| RAD51B_CpG_18 | 0.045 | 0.466 |  | 0.057 | 0.368 |
| RAD51B_CpG_20 | 0.077 | 0.213 |  | 0.102 | 0.105 |

Bold values indicated *p* < 0.05.
