# Supplementary material for: The Association Between Breast Cancer and Blood-Based Methylation of CD160, ISYNA1 and RAD51B in the Chinese Population
Source: Front Genet. 2022 Jun 9;13:927519. doi: 10.3389/fgene.2022.927519 (PMC9261985; doi:10.3389/fgene.2022.927519)
Supplement: Supplementary file 2 [file DataSheet2.docx]

**Supplementary Figure**

**A**

GGATTCCTGAATGCCCATGGAAGAAAAGCGAGCATGCTGGTCTGG**CG**GGCAGAAATAACCTTCTCCCCAGGCTCCAGAGGGCAGAGGACATTTCCCTCCAAAAGGCAAGTTTCTCAGGGACCTCATGAGAAGCCTGTCCTCACCTGAGCAGTTCTGTCAACCATCCCATCCCTTCACCTGGACCCCAGGTCCCCAGCAGGCCTTTTTACCT**CG**AGACAGAAGTTCTTCTTTCTTGTTCTCAGACTGGGGGCTCA**CG**TTGAAATGGCTCTCAGTCCTCAGATTGTCCTTGCATCTTGTTAAAACCCTATCTCTCCAGTTT**CG**TCATTGCTCACACTTTCATAGCTGTTATTATATT**CG**CTCTTTGAAAACCACAGAATGGGTTGTTAGAAGTCATAGATCTTC**CG**CCACCCCCACTGTGGATTTTTTTCTCCTTTCTCTCCTGCAACTGGTCCTCCATCTCCTTCTCCTTTCCTCTCCCTTGTTTTGTCTTTTTGGTGT

**B**

GGCCTGTGACCTGACTCTGCCTCCCTCTACTTCCC**CG**ACTCTTGAG**CG**CCCAGCTTCAGTCCCATCCCCTCTGCCCATGGTCATCACCCCAACTCCCAGGGAGAGCCCAGAACCTGGAGCCT**CG**CTCTGCCCCCAGTTCTCAAAGGTGGGGCTAGATGGGGG**CG**GGGACGGAACAAGACCACAGGGTGGAGAGTGTGGTGGCAGAAGGCCAAAGAGGGAATCTGAGACAAGCTACCAAAAACCAAAACCAGCTTCCATTTATTTATGGGCCCCACCCCCTGA**CG**TCCTGGGGAGAGGGGCCCCCCAAAGAGCCTTTCTCTCTGGGACAAAATACAAATGGCAGGAAGGGTCCATACAGAAAAGAA**CG**TCTCTGAGGTCCTTGCGTTTTTAATAAAATGGTAAGAGTCTTT**CG**TGTGGGGCCTCCCAGCTGGGCAAGCCTTGATTGGCCAGGAGT

**C**

GTCTTGAGTGGAGACAGGGCTCCCCTGAGGCC**CGCG**ATGAGGAGTGGG**CG**CAGAGGGAGGGAGGGAGAAGAGGGTAGTCAGGGATCAGAGGGGTGGCTGGGGAAGGGGACACACACACTGGGGCAAT**CG**GAGAGCAGGGAGGATGGAGGCGGAAGGAGAGAGGGTTAGGATGAG**CG**GAT**CG**GGAAGGAACATGG**CG**AAGCAAAGCTGAGCTCAGCAGCAGATACTTCCTT**CG**CCACACACTCCC**CG**CACTGGAGAAGGAAGCC**CG**TGGAGGCCT**CG**CTCTCACTGGGG**CGCG**TGTGGGCAAACAAGCCTGCAGTCCAGCCAGAAG**CG**GCCTTCCCACCACACAACTTCCTCCTGTGGA**CG**CTAACACTGACCGATGTGGCCCCTTCTCTGCAATCTCTGTGCAATGGAG**CG**GCTGATACGACTTCCAGCCTACA**CG**GGAGCACACAGAAGCACAGTGGTTGAGGGTAGACTCTGAGTC

**Supplementary Figure 1**. Sequences of the CD160, ISYNA1 and RAD51B amplicons. **(A)** The sequence of the CD160 amplicon examined by the EpiTyper assay (chr1: 145,715,317-145,715,815, build 37/hg19, defined by the UCSC Genome Browser). The EpiTyper assay determined the methylation levels of 7 CpGs in this amplicon and yielded 6 distinguishable mass peaks. The CpG sites that could be measured are in bold, cg20975414 (CD160_CpG_4) and cg12832565 (CD160_CpG_6) are in bold and underlined. **(B)** The sequence of the ISYNA1 amplicon examined by the EpiTyper assay (chr19: 18,545,150-18,545,604, build 37/hg19, defined by the UCSC Genome Browser). The EpiTyper assay determined the methylation levels of 9 CpGs in this amplicon and yielded 7 distinguishable mass peaks. The CpG sites that could be measured are in bold, cg22161383 (ISYNA1_CpG_4) is in bold and underlined. **(C)** The sequence of the RAD51B amplicon examined by the EpiTyper assay (chr14:68,830,515-68,830,993, build 37/hg19, defined by the UCSC Genome Browser). The EpiTyper assay determined the methylation levels of 20 CpGs in this amplicon and yielded 15 distinguishable mass peaks. Each of the 2 peaks contains two CpG sites including RAD51B_CpG_1 and RAD51B_CpG_2, RAD51B_CpG_13 and RAD51B_CpG_14, whereas each of the other 13 peaks contains only one single CpG site. In addition, the mass peaks show the same methylation level of RAD51B_CpG_7 and RAD51B_CpG_8, and thus the two CpG sites are presented as RAD51B_CpG_7.8 in the manuscript. The CpG sites that could be measured are in bold, cg13803234 (RAD51B_CpG_7) and cg10975863 (RAD51B_CpG_13) are in bold and underlined.
